# Lignocaine/phenylephrine nasal spray vs. placebo for the pain and distress of nasogastric tube insertion in children: a study protocol for a randomized controlled trial

**DOI:** 10.1186/s13063-015-0547-y

**Published:** 2015-01-27

**Authors:** Simon S Craig, Robert W Seith, John A Cheek, Adam West, Kathryn Wilson, Diana Egerton-Warburton

**Affiliations:** Emergency Department, Monash Medical Centre, Monash Health, Clayton, Australia; School of Clinical Sciences at Monash Health, Monash University, Clayton, Australia; Murdoch Children’s Research Institute, Parkville, Australia; Emergency Department, Royal Children’s Hospital Melbourne, Parkville, Australia

**Keywords:** Pain/prevention and control, Anesthetics, Local/administration and dosage, Child, Preschool, Intubation, Gastrointestinal

## Abstract

**Background:**

Patients and clinicians consistently rate insertion of a nasogastric tube (NGT) as one of the most painful and distressing emergency department procedures. Despite this, surveys of emergency clinicians suggest that provision of adequate procedural analgesia is often inconsistent and suboptimal. While many studies have demonstrated the effectiveness of various interventions to reduce pain and distress in adults, there have been few studies in the pediatric population. There are currently no studies comparing the effectiveness of a local anesthetic nasal spray for the prevention of the pain and distress associated with NGT insertion in children. This study aims to compare the analgesic efficacy of a proprietary preparation of lignocaine/phenylephrine nasal spray and placebo for this indication.

**Methods/Design:**

This is a prospective, randomized, controlled, double-blind superiority trial of 100 children aged 6 months to 5 years weighing at least 6 kg in whom a nasogastric tube is planned to be inserted. These children will be randomized to either intranasal lignocaine/phenylephrine or placebo. Pain severity is the primary outcome measure and will be measured utilizing the Face, Legs, Arms, Cry and Consolability (FLACC) pain severity rating scale. An independent staff member not involved in inserting the NGT and the child’s parents or carer will also record pain and distress on a visual analog scale (VAS). FLACC scores and VAS scores will be presented as median and interquartile range (IQR). Non-normally distributed scores will be compared using a Wilcoxon rank-sum test. Categorical data will be analyzed using Fisher’s exact test. Adverse events will be described as type and incidence.

**Discussion:**

Previous studies on NGT insertion have not focused on the pediatric population. This study aims to establish the effectiveness of a simple intranasal spray of lignocaine/phenylephrine in children undergoing NGT insertion. A positive result of this study would provide evidence of an effective intervention in a procedure considered by many to be very painful and distressing.

**Trial registration:**

Australian New Zealand Clinical Trials Registry (ANZCTR). ACTRN12614000092695, registered on 23 January 2014.

**Electronic supplementary material:**

The online version of this article (doi:10.1186/s13063-015-0547-y) contains supplementary material, which is available to authorized users.

## Background

Insertion of a nasogastric tube is consistently rated by patients and clinicians as one of the most painful and distressing procedures performed in emergency departments [1]. Despite this, surveys of emergency physicians and trainees suggest that provision of adequate procedural analgesia is inconsistent and suboptimal in many instances [2,3].

There have been many studies examining the effect of various interventions to reduce the pain and distress of nasogastric tube insertion in adults. Most published interventions in adult patients involve some sort of local anesthetic administration with the use of topical nasal spray [2,4-6], nebulization [7,8], or a gel [9]. These are all more effective than a placebo; however, the relative superiority of one method over another is yet to be clearly demonstrated. More recently, intranasal ketamine (50 mg) was also found to be more effective than a placebo in reducing the discomfort of nasogastric tube insertion in 72 adult patients [10].

The pediatric literature is limited, with only one study published in 2009. Babl and colleagues compared nebulized lignocaine vs. placebo in a pediatric emergency department population. The study was terminated early, before the intended 52 subjects had been enrolled, because of staff concern about the level of patient distress during nebulization [11]. Children appear less able to tolerate nebulized therapy.

Interestingly, children also appear to have different responses to topical local anesthetic application for painful mucosal conditions. A recently published trial by Hopper and colleagues demonstrated no difference in oral intake in children with painful infectious mouth ulcers when administered viscous lignocaine compared with placebo [12].

There are currently no studies comparing the effectiveness of a local anesthetic nasal spray for the prevention of pain and distress associated with nasogastric tube insertion in children. This study aims to compare the analgesic efficacy of a proprietary preparation of lignocaine/phenylephrine nasal spray and placebo for this indication. It is hypothesized that intranasal lignocaine/phenylephrine spray will be more effective than intranasal placebo in the prevention of pain and distress associated with nasogastric tube insertion in children.

## Methods/Design

This is a prospective, randomized, controlled, double-blind superiority trial. The trial will be conducted in the emergency department of Monash Medical Centre, a tertiary referral hospital at which the Pediatric ED has an annual census of over 27,000 patients. Participant inclusion and exclusion criteria are shown in Table 1.

**Table 1:** **Inclusion and exclusion criteria for the study**

**Inclusion criteria**	**Exclusion criteria**
Children aged 6 months to 5 years and weighing at least 6 kg body mass	Inability to gain informed consent from parent or guardian
	Indication for an urgent insertion of a nasogastric tube
Planned to have a nasogastric tube inserted as part of their emergency department treatment	Accompanying adult is non-English speaking and no interpreter service is available
	Child or parent has an allergy to lignocaine or phenylephrine
	Aberrant nasal anatomy
	Acute or chronic nasal problems or nasal trauma that may preclude adequate administration or absorption of intranasal medication.
	Cardiovascular disease/congenital heart disease – specifically hypertension, severe bradycardia, conduction disturbances, and digitalis intoxication
	Known hepatic or renal impairment
	Asthma (particularly sulfite-sensitive asthma)
	Genetic predisposition to malignant hyperthermia
	Preexisting abnormal neurological conditions
	Child is taking medications known to interact with CoPhenylcaine Forte™ (antiarrhythmic drugs, suxamethonium, phenytoin, antidepressants, propranolol, citicoline)

### Randomization and storage

One hundred spray bottle numbers will be randomly allocated to either lignocaine/phenylephrine or placebo. Randomization will be made following a block randomization table as described by Altman and Bland (1999) [13]. Each spray bottle will be manufactured as allocated, and this number will be recorded on the patient prescription accompanying the spray bottle.

### Study medication

CoPhenylcaine Forte Nasal Spray™ (ENT Technologies Pty Ltd., Hawthorn East, Melbourne, Australia) is a proprietary product that contains the active ingredients of lignocaine hydrochloride 50 mg/ml (5% lignocaine) and phenylephrine hydrochloride 5 mg/ml (0.5% phenylephrine). It is administered by a pump-actuated topical spray. The nozzle spray attachment provides 100 μl per spray. This results in each spray of active medication delivering 5 mg lignocaine and 0.5 mg phenylephrine. A preparation of sodium chloride 0.9% will be used as placebo.

Lignocaine/phenylephrine spray bottles will be made by transferring 2.5 ml of CoPhenylcaine Forte into a spray bottle. This will then be labeled and kept in a sealed plastic bag until use. Placebo spray bottles will be made by transferring 2.5 ml of sodium chloride 0.9% for irrigation into a spray bottle. This will again be labeled and kept in a sealed plastic bag until use.

Spray bottle numbers, constituents, their batch number, and expiry will be recorded by sterile manufacturing and clinical trials pharmacy. A prescription marked with the corresponding spray bottle number will be included in each bag—for return to the pharmacy for study accountability.

The study spray bottles will be given an expiry of 1 month from manufacture. Twenty spray bottles will be prepared monthly by the pharmacy. Each month unused spray bottles will be deemed expired and destroyed by the pharmacy. Spray bottle numbers not used will be documented and later re-made (with the same allocation) until all 100 spray bottle numbers are accounted for. If recruitment of patients is in excess of five per week, the spray bottle number production will be reviewed and may increase up to a maximum of eight per week.

### Measurement tools

#### Pain severity rating scale (FLACC)

The primary outcome measure will be the pain rating using the Faces, Legs, Activity, Cry and Consolability (FLACC) Scale during the procedure. The FLACC score was originally designed for assessment of postoperative pain in young children and has been recommended in various reviews as a procedural pain score for preverbal and early verbal children [14]. It comprises five separate items (face, legs, activity, cry, and consolability), each of which is scored from zero to two. The five scores are then added to arrive at a score out of ten.

Scores will be assessed by a member of the staff not directly involved in the insertion of the nasogastric tube at four times during the study: (1) In the ED cubicle prior to study drug administration (baseline); (2) in the procedure room when the child is positioned for NGT insertion (prior to insertion attempts); (3) during the final NGT insertion attempt (whether successful or unsuccessful); (4) once the child has been returned to the ED cubicle.

### Observer pain severity rating

Visual Analog Scale (VAS): An observer (a staff member not directly involved in the procedure) will be asked to record impressions of pain and distress by marking on a standard 100-mm line labeled ‘no pain’ or ‘no distress’ at the left hand end and ‘severe pain’ or ‘severe distress’ at the right hand end. Parents will also be asked to complete the same visual analog scales for pain and distress.

### Other information to be recorded

Other information to be recorded includes the experience and confidence of the proceduralist, time from nasal spray administration to successful tube insertion, number of attempts required to insert the nasogastric tube, procedural complications (of the nasal spray and/or of nasogastric tube insertion, such as epistaxis and tube misplacement), and methods used to confirm nasogastric tube placement. The clinicians performing the procedure will record the difficulty of nasogastric tube insertion on a VAS and will be asked to record their judgment as to whether placebo or lignocaine/phenylephrine was administered.

### Procedure

The parents or carers of eligible patients will be approached for enrollment in the study. Written, informed consent will be obtained. Parent information statements include references to possible side effects, including tremor, palpitations, or a bitter taste in the mouth.

On obtaining consent, baseline measurement of the pain score will be recorded. The study medication will be obtained from the next numbered study pack and administered intranasally using the pump-actuated spray. Children weighing 6 to 12 kg will be administered one spray to each nostril, while children weighing over 12 kg will be administered two sprays to each nostril.

**Figure 1:**
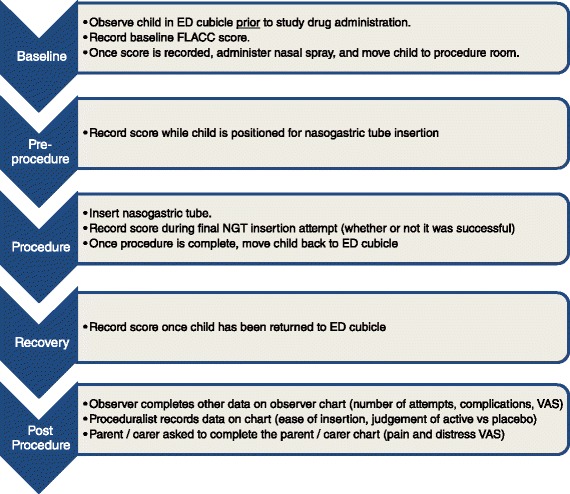
**Study flowchart.**

Figure 1 provides an overview of the study, indicating the times of medication administration and data recording.

### Outcome measures

The primary outcome measure is the FLACC score recorded during the final attempt at nasogastric tube insertion. Secondary outcome measures will include the VAS scores for pain, distress, ease of tube insertion, number of attempts required, and procedural complications.

### Statistical analysis

Baseline variables such as sex and age will be presented as the number and percentage or median with interquartile range and be compared using either chi-square or Mann–Whitney tests as appropriate. FLACC and VAS scores will be presented as median and interquartile range (IQR). Non-normally distributed scores will be compared using a Wilcoxon rank-sum test and normally distributed data using an independent t-test. Categorical data will be analyzed using Fisher’s exact test. Adverse events will be described as type and incidence. Analysis will be intention to treat.

### Sample size

Assuming a standard deviation of 2.5, an α of 0.05, and a power of 90%, 35 patients per treatment arm are required to demonstrate a 2-point difference in the FLACC score. This difference has previously been considered the minimally clinically significant difference using this scoring system [15]. Allowing for attrition and other factors, a total of 100 patients is required (50 in each group).

### Ethical considerations

This clinical trial has the Human Research Ethics Committee (HREC) approval of Monash Health HREC Committee B [see Additional file 1]. The trial is registered with the Australian New Zealand Clinical Trials Registry (ANZCTR), ACTRN12614000092695. The Monash Health Drug and Therapeutics Committee reviewed the trial protocol. A trial safety monitoring committee has been incorporated into the study design to review adverse events and therapeutic efficacy in the trial. The study has also been registered with the Australian Government Therapeutic Goods Administration Clinical Trials Notification (CTN) Scheme (trial no. 2014/0367, protocol no. 13410A), and complies with the relevant SPIRIT statement (Additional file 1) and WHO checklist (Additional file 2).

## Discussion

### Scope of the trial

While it is clear that there are many effective options to reduce the pain and distress associated with nasogastric tube insertion in adults, there is very little published literature in the pediatric setting. Previous studies have demonstrated that children may not tolerate treatments that are easily administered to adults (such as nebulization) and do not respond predictably to administration of topical local anesthetics. This study aims to determine the effectiveness of a simple nasal spray of lignocaine and phenylephrine in children undergoing nasogastric tube insertion.

### Strengths

To date, there is little literature to guide procedural pain management in children undergoing nasogastric tube insertion. If positive, this blinded, randomized study will provide useful information – including the effect size – to assist healthcare providers conduct the procedure with minimal pain and distress for the child.

### Limitations

The results of this study will only apply to children aged 6 months to 5 years without any comorbid disease. It is unclear whether the results can be extrapolated to older children or those with significant comorbidities.

Although our primary outcome measure – the FLACC score – is recommended for the measurement of procedural pain and distress in young children [16], it may have limited ability to discriminate during highly painful and distressing parts of a procedure [11].

### Summary

Nasogastric tube insertion is a commonly performed procedure in pediatric emergency medicine. To date, no studies have been able to demonstrate effective methods to reduce the pain and distress associated with the procedure in children. This study, if positive, would provide evidence of an effective method to reduce distress for young children having nasogastric tube insertion in the ED.

## Trial status

Recruitment commenced in July 2014 for the 100 participants needed for the trial. It is anticipated that recruitment will be completed by late 2015.

## Additional files

Additional file 1:
**SPIRIT statement.**
Additional file 2:
**WHO Checklist.**

## Abbreviations

ANZCTR: Australian New Zealand Clinical Trials Registry
CTN Scheme: Clinical trials notification scheme
ED: Emergency department
ENT: Ear, nose, and throat
FLACC scale: Faces, Legs, Activity, Cry, and Consolability scale
HREC: Human Research Ethics Committee
IQR: Interquartile range
NGT: Nasogastric tube
VAS: Visual analog scale
