# Supplementary material for: Lignocaine/phenylephrine nasal spray vs. placebo for the pain and distress of nasogastric tube insertion in children: a study protocol for a randomized controlled trial
Source: Trials. 2015 Jan 27;16:30. doi: 10.1186/s13063-015-0547-y (PMC4318482; doi:10.1186/s13063-015-0547-y)
Supplement: Additional file 2: — WHO Checklist. [file 13063_2015_547_MOESM2_ESM.docx]

|  | **Information**[**^32^**](http://www.spirit-statement.org/spirit-statement/references#32) |
| --- | --- |
| Primary registry and trial identifying number | Australian New Zealand Clinical Trials Registry (ANZCTR) ACTRN12614000092695 |
| Date of registration in primary registry | 23/01/2014 |
| Secondary identifying numbers | N/A |
| Source(s) of monetary or material support | Morson Taylor Research Award  Emergency Medicine Research Foundation  c/o Australasian College for Emergency Medicine |
| Primary sponsor | Emergency Medicine Research Foundation |
| Secondary sponsor(s) | N/A |
| Contact for public queries | Simon Craig, FACEM  Emergency Department, Monash Medical Centre , Clayton, Victoria, Australia  61 3 9594 2707  [Simon.craig@monashhealth.org](mailto:Simon.craig@monashhealth.org) |
| Contact for scientific queries | Simon Craig, FACEM  Emergency Department, Monash Medical Centre , Clayton, Victoria, Australia  61 3 9594 2707  [Simon.craig@monashhealth.org](mailto:Simon.craig@monashhealth.org) |
| Public title | Reducing pain and distress associated with nasogastric tube insertion in young children. |
| Scientific title | **A randomized double-blind controlled trial of lignocaine / phenylephrine nasal spray vs placebo for pain and distress of nasogastric tube insertion in children.** |
| Countries of recruitment | Australia |
| Health condition(s) or problem(s) studied | Procedural pain and distress associated with nasogastric tube insertion. |
| Intervention(s) | Placebo: Sodium chloride 0.9% nasal spray  Intervention: Lignocaine / phenylephrine nasal spray |
| Key inclusion and exclusion criteria | Inclusion criteria:  Children aged six months to five years and weighing at least 6 kg body mass.  Planned to have a nasogastric tube inserted as part of their emergency department treatment.  . . Exclusion criteria:  Child or parent has an allergy to lignocaine or phenylephrine  Contraindications to nasal medication administration  Coexisting medical conditions: cardiovascular disease / congenital heart disease – specifically hypertension, severe bradycardia, conduction disturbances and digitalis intoxication. Known hepatic or renal impairment, asthma (particularly sulfite-sensitive asthma), genetic predisposition to malignant hyperthermia. Pre-existing abnormal neurological conditions.  Child is taking medications known to interact with active medications (antiarrhythmic drugs, suxamethonium, phenytoin, antidepressants, propranolol, citicoline) |
| Study type | Interventional Allocation: randomized Intervention model: parallel assignment Masking: double blind (subject, caregiver, investigator, outcomes assessor) Primary purpose: prevention of pain and distress Phase III |
| Date of first enrolment | July 2014 |
| Target sample size | 100 |
| Recruitment status | Recruiting |
| Primary outcome(s) | Pain severity measured utilizing the Face, Legs, Arms, Cry and Consolability (FLACC) pain severity rating scale |
| Key secondary outcomes | Difficulty of nasogastric tube insertion., number of attempts required to insert the nasogastric tube, procedural complications (of the nasal spray and/or of nasogastric tube insertion, such as epistaxis and tube misplacement), and methods used to confirm nasogastric tube placement. |
